# Unveiling the Leishmanicidal Mechanism of 4‐Nerolidylcatechol Isolated From *Piper peltatum* Against *Leishmania infantum*

**DOI:** 10.1002/cbdv.202503244

**Published:** 2026-01-10

**Authors:** Fabiana Brusco Lorenzetti, Rodolfo Bento Balbinot, Danielle Lazarin‐Bidóia, Viviane dos Santos Faiões, Eduardo Caio Torres‐Santos, Celso Vataru Nakamura, Tânia Ueda‐Nakamura, Diógenes Aparício Garcia Cortez, Benedito Prado Dias Filho

**Affiliations:** ^1^ Laboratório de Inovação Tecnológica no Desenvolvimento de Fármacos e Cosméticos Universidade Estadual de Maringá Maringá Paraná Brazil; ^2^ Laboratório de Bioquímica de Tripanosomatídeos Instituto Oswaldo Cruz—FIOCRUZ Rio de Janeiro Rio de Janeiro Brazil

**Keywords:** action mechanism, drug discovery, phytochemistry, terpenoids, ultrastructural evaluation

## Abstract

The present study assessed the leishmanicidal activity of 4‐nerolidylcatechol (4‐NC) (**1**) isolated from *Piper peltatum* leaves against promastigotes and amastigotes of *Leishmania amazonensis*, *L. braziliensis*, and *L. infantum*. The plant was fractionated, leading to the isolation and chemical identification of 4‐NC (**1**). Biochemical techniques were subsequently employed to investigate the mechanism of action of both the extract and the compound. The induction of cellular apoptosis through reactive oxygen species production appears to be a predominant mechanism of action. In addition, both 4‐NC (**1**) and the hydroethanolic extract showed minimal toxicity to human cells, even at higher concentrations. To our knowledge, this study represents the first report highlighting the leishmanicidal properties of 4‐NC (**1**).

## Graphical abstract

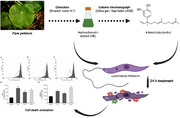

## 1 Introduction

Leishmaniasis is a parasitic disease caused by protozoan parasites of the genus *Leishmania*, which comprises more than 30 species, about twenty of which are known to be pathogenic to humans [1]. These parasites are transmitted through the bites of infected female sandflies of the genera *Phlebotomus* (in the Old World) and *Lutzomyia* (in the New World). The disease is endemic in nearly 100 countries, primarily in tropical and subtropical regions, such as parts of Africa, Asia, and Latin America. It disproportionately affects impoverished populations living in areas with limited access to healthcare and poor urban infrastructure [2]. Classified as a neglected tropical disease (NTD), leishmaniasis affects over 12 million people worldwide, with an estimated 700 000 to 1 million new cases each year. Despite its significant public health burden, leishmaniasis remains underreported and underfunded, posing a persistent challenge in endemic areas [3].

*Leishmania* parasites exhibit a digenetic life cycle, alternating between promastigote forms in the sandfly vector's midgut and intracellular amastigote forms within the phagocytic cells of the mammalian host [4]. Depending on the parasite species, its virulence, and the host's immune response, leishmaniasis can manifest in diverse clinical forms, ranging from small skin lesions to extensive ulcerations in cutaneous (CL) and mucocutaneous leishmaniasis (MCL), or as potentially fatal systemic infections in visceral leishmaniasis (VL), also known as kala‐azar [5, 6].

Current therapeutic options for leishmaniasis include miltefosine, amphotericin B (and its liposomal form), pentavalent antimonials, paromomycin, azoles, and pentamidine [7, 8]. However, these therapies face several limitations, such as high toxicity, severe side effects, variable efficacy depending on the *Leishmania* species and geographical region, and the growing emergence of drug resistance [9]. Miltefosine, the only approved oral agent, is further constrained by gastrointestinal toxicity, teratogenicity potential, and reduced effectiveness against some forms of tegumentary leishmaniasis [10]. Although liposomal amphotericin B reduces toxicity, its high‐cost limits accessibility in low‐resource settings. Furthermore, *Leishmania* parasites may persist in host tissues even after clinical recovery, increasing the risk of relapse or post‐kala‐azar dermal leishmaniasis (PKDL) [11].

Considering these limitations, the search for alternative treatments is essential to improve therapeutic outcomes. In this context, new studies have focused on developing innovative therapies based on natural products, especially medicinal plants, as potential sources of novel drugs. Plant‐derived compounds offer a wide variety of bioactive molecules, such as alkaloids, flavonoids, steroids, and tannins, many of which exhibit promising pharmacological properties. This approach aims to identify substances that are less toxic and more accessible to low‐income populations, which are often the most affected by leishmaniasis [12].

Due to the limited therapeutic efficacy and high toxicity of current medications, the exploration of traditional medicinal knowledge has emerged as a promising alternative, particularly regarding bioactive compounds derived from plants. Indeed, plants may serve as valuable sources of safe and effective new drugs for the treatment of infectious diseases, stimulating significant investment from the pharmaceutical industry in plant‐based research [13, 14, 15].

Several studies have demonstrated the biological activity of plant species of the *Piper* genus, such as the ethanol extract of *P. betle* leaves [16], the compound 2′,6′‐dihydroxy‐4′‐methoxychalcone isolated from the inflorescence of *P. aduncum* [17], the extracts from leaves of *P. rusbyi* [18], and the crude and chloroform extract of *P. reginelli* [19]. Within the genus *Piper*, the species *P. peltatum* has demonstrated notable pharmacological potential and various recognized bioactivities. Commonly known as “monkey's hand,” “Capeba do Norte,” or “long pepper,” it is traditionally used to treat inflammation and ulcers and as a hepatoprotective agent [20]. Reported activities include inhibition of *Plasmodium falciparum* growth [21], anti‐inflammatory effects [22], and inhibition of myotoxin I phospholipase activity from *Bothrops* venom [23]. It has also shown in vitro larvicidal activity against *Aedes aegypti* [24].

Antimicrobial effects have also been attributed to *P. peltatum* extracts. Mongelli et al. [25] observed partial inhibition of *Staphylococcus aureus* using decoctions from the plant's aerial parts. Additionally, *P. peltatum* is recognized for its antioxidant properties. According to Pasqualoto et al. [26], its antioxidant activity is related to the oxidation of the alkenyl side chain of 4‐nerolidylcatechol (4‐NC) (**1**), the plant's main compound. This compound, first isolated by Kijjoa et al. [27], exhibits various pharmacological properties, including antitumor activity [28, 29], inhibition of androgen‐independent prostate cancer cells [30], antimalarial [31], antidermatophytic [32], and schistosomicidal effects [33].

Given the urgent need for new, safer, more effective, and accessible therapeutic strategies, the aim of the present study was to perform a phytochemical analysis and to evaluate the activity of the hydroalcoholic extract and pure compound from the leaves of *P. peltatum* against the promastigote and amastigote forms of *Leishmania amazonensis*, *L. braziliensis*, and *L. infantum*, as well as to assess the cytotoxicity of the pure compound 4‐NC. Additionally, morphological, ultrastructural, and biochemical alterations induced by these compounds in the promastigote forms were analyzed.

## 2 Results and Discussion

### 2.1 Phytochemical Study

**FIGURE 1:**
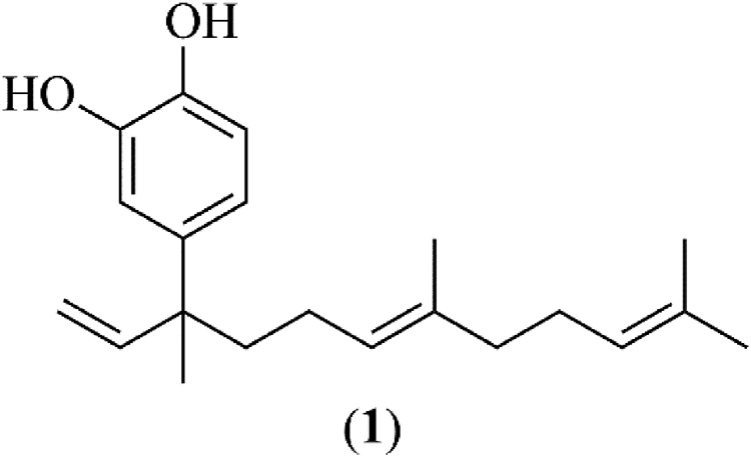
Chemical structure of 4‐nerolidylcatechol (4‐NC) (**1**).

Phytochemical study of *P. peltatum* leaves performed by bioassay‐guided fractionation (Table 1) led to the isolation of the bioactive compound 4‐NC (**1**) (Figure 1). The compound (**1**) was identified as 4‐NC on the basis of spectroscopic analyses (UV, ^1^H NMR, ^13^C NMR, ^1^H–^1^H COSY, gHSQC, and gHMBC) and by comparison with data from literature [27] (Supporting information).

**TABLE 1:** Ultrashort summary of extraction and isolation of Compound **1**.

Step	Key information	Result
Extraction	Leaves (700 g) → EtOH/H_2_O 9:1	HE (26.5 g)
Main Fractions	Hexane (HF), Hexane/CH_2_Cl_2_ (HDF), CH_2_Cl_2_ (DF), CH_2_Cl_2_/EtOAc (DEAF), EtOAc, MeOH	—
DEAF	71 Subfractions	DEAF 30 → Compound 1 (12.5 mg)
HF	110 Subfractions; HF 40–54 (128 mg)	→ Compound 1 (25.0 mg)
HDF	100 Subfractions; HDF 32–44 + 56–60	→ Compound 1 (17.0 mg)

*Note*: HF, HDF, DF, DEAF: fractions obtained by vacuum column chromatography using increasing polarity solvent systems; subfractions were combined on the basis of the TLC similarity; yields refer to isolated mass of purified Compound 1 after final chromatographic steps.

Abbreviations: 4‐NC, 4‐nerolidylcatechol; DF, dichloromethane fraction; EtOAc, ethyl acetate; EtOH, ethanol; HE, hydroethanolic extract; HF, hexane fraction; MeOH, methanol.

4‐NC (**1**) is a compound commonly isolated from plants of the *Piper* genus, especially *P. peltatum*, *P. marginatum*, and *P. umbellatum*, and it is reported as the major constituent in extracts obtained from these species [33, 34]. Despite a range of biological activities described in the literature its antileishmanial potential has not yet been described, to the best of our knowledge. Therefore, we evaluated the activity of both extracts and pure 4‐NC (**1**) against different *Leishmania* species.

### 2.2 Biological Assays

This study began with an initial screening of the antiproliferative activity of the hydroethanolic extract (HE) from the leaves of *P. peltatum*, its solvent‐partitioned fractions, and the isolated compound 4‐NC (**1**) against promastigote forms of *L. amazonensis*, *L. braziliensis*, and *L. infantum*. Promastigotes were selected for this preliminary evaluation due to their ease of manipulation under in vitro conditions and because they represent the standard first step in antileishmanial screening assays.

In the promastigote assays, HE consistently exhibited the highest antiparasitic activity among all tested samples. Against *L. infantum*, HE displayed an inhibitory concentration for 50% of parasites (IC_50_) of 5.6 µg mL^−1^, whereas for *L. braziliensis* and *L. amazonensis*, the IC_50_ values were 10.6 and 7.6 µg mL^−1^, respectively (Table 2). The isolated compound 4‐NC (**1**) showed lower activity compared to HE, with IC_50_ values of 17.4, 44.6, and 17.8 µg mL^−1^ against *L. infantum*, *L. braziliensis*, and *L. amazonensis*, respectively. Among the fractions, the hexane/dichloromethane (1:1 v/v) fraction (HDF), dichloromethane fraction (DF), and dichloromethane/ethyl acetate (1:1 v/v) fraction (DEAF) exhibited moderate antipromastigote activity, whereas hexane fraction (HF), methanolic fraction (MF), and ethyl acetate fraction (EAF) consistently showed the lowest activity across all species. These findings are consistent with previous reports of antileishmanial activity in extracts from species of the *Piper* genus, including *P. rusbyi* and *P. marginatum*, which demonstrated activity against promastigotes of multiple *Leishmania* species [18, 35].

**TABLE 2:** Antileishmanial activity of hydroethanolic extract, fractions and 4‐nerolidylcatechol (4‐NC) (**1**) isolated from *Piper peltatum* against promastigotes forms of *Leishmania* spp.

Samples	*L. infantum*	*L. amazonensis*	*L. braziliensis*
	IC_50_ (µg mL^−1^)	IC_50_ (µg mL^−1^)	IC_50_ (µg mL^−1^)
HE	5.6	7.6	10.6
4‐NC (**1**)	17.4	17.8	44.6
HF	39.6	17.5	>50.0
HDF	30.3	8.8	44.5
DF	30.5	16.0	35.1
DEAF	36.6	28.2	>50.0
EAF	>50.0	>50.0	>50.0
MF	>50.0	>50.0	>50.0
Pentamidine	3.3	2.84	0.9

*Note*: Data are expressed as mean from at least three independent experiments.

Abbreviations: 4‐NC (**1**), 4‐nerolidylcatechol; DEAF, dichloromethane/ethyl acetate (1:1 v/v) fraction; DF, dichloromethane fraction; EAF, ethyl acetate fraction; HDF, hexane/dichloromethane (1:1 v/v) fraction; HE, hydroethanolic extract; HF, hexane fraction; IC_50_, inhibitory concentration for 50% of parasites; MF, methanolic fraction.

On the basis of the promastigote screening, HE, the DEAF fraction (4‐NC (**1**) isolated from this fraction), and 4‐NC (**1**) were selected for further evaluation against intracellular amastigotes, the clinically relevant form of the parasite responsible for disease progression in infected individuals. In these assays, HE once again demonstrated strong activity. The IC_50_ values were 9.3 µg mL^−1^ for *L. infantum*, 1.7 µg mL^−1^ for *L. braziliensis*, and 1.5 µg mL^−1^ for *L. amazonensis*. The isolated compound 4‐NC (**1**) was also effective, with IC_50_ values of 11.5, 8.3, and 7.8 µg mL^−1^, respectively. The DEAF fraction presents IC_50_ values of 10.6 µg mL^−1^ against amastigotes of *L. infantum*, 7.8 µg mL^−1^ against *L. amazonensis*, and 8.1 µg mL^−1^ against *L. braziliensis*. Fractions that exhibited low activity during the promastigote screening (HF, EAF, and MF) were not evaluated in amastigote assays.

Cytotoxicity assays were performed in macrophages to assess the safety profile of the most active samples. The HE, DEAF, and 4‐NC (**1**) showed cytotoxic concentration for 50% of the cells (CC_50_) values of 30.7, 42.3, and 47.4 µg mL^−1^, respectively. These cytotoxicity data were subsequently integrated with antiparasitic activity to calculate selectivity index (SI = CC_50_/IC_50_) values. The HE presented SI values of 3.3, 18.1, and 20.5 for *L. infantum*, *L. braziliensis*, and *L. amazonensis*. The compound 4‐NC (**1**) displayed an SI of 4.1, 5.7, and 6.1, respectively. The DEAF fraction showed the highest SI for *L. amazonensis*, with a value of 5.4; for *L. braziliensis*, the SI value was 5.2 and for *L. infantum*, the SI value achieved was 4.0. All tested fractions exhibited SI values greater than 1, indicating lower toxicity toward mammalian cells compared with parasites (Table 3).

**TABLE 3:** Antiproliferative activity of hydroethanolic extract, fraction, and compound isolated of *Piper peltatum* against amastigotes forms of *Leishmania* spp. and cytotoxic activity on macrophages.

Samples	Macrophages	*L. infantum*		*L. amazonensis*		*L. braziliensis*	
		Amastigotes		Amastigotes		Amastigotes	
	CC_50_ (µg mL^−1^)	IC_50_ (µg mL^−1^)	SI	IC_50_ (µg mL^−1^)	SI	IC_50_ (µg mL^−1^)	SI
HE	30.7	9.3	3.3	1.5	20.5	1.7	18.1
DEAF	42.3	10.6	4.0	7.8	5.4	8.1	5.2
4‐NC (**1**)	47.4	11.5	4.1	7.8	6.1	8.3	5.7
Pentamidine	5.03	2.3	2.2	1.1	4.6	1.5	4.8

*Note*: Data are expressed as mean from at least three independent experiments.

Abbreviations: 4‐NC (**1**), 4‐nerolidylcatechol; CC_50_, cytotoxic concentration for 50% of cells; DEAF, dichloromethane/ethyl acetate (1:1 v/v) fraction; HE, hydroethanolic extract; IC_50_, inhibitory concentration for 50% of parasites; SI, selectivity index (CC_50_/IC_50_).

The consistently higher activity of HE compared to 4‐NC (**1**) may be explained by synergistic interactions among the chemical constituents of the crude extract, a phenomenon frequently observed in natural products derived from complex phytochemical mixtures [36]. Together, these findings demonstrate that HE, DEAF, and 4‐NC (**1**) exhibit promising antileishmanial properties, with activity confirmed in both promastigote and amastigote forms and supported by favorable selectivity profiles.

Differences in antileishmanial activity observed between promastigote and amastigote forms can be attributed to intrinsic biological and physiological distinctions between these developmental stages. Promastigotes are extracellular and remain freely exposed in the culture medium, allowing the tested substances to act directly on the parasite surface and intracellular structures without physical barriers. In contrast, amastigotes reside within parasitophorous vacuoles inside macrophages, meaning that compounds must first cross the host cell membrane and then penetrate the vacuolar compartment before reaching the parasite. This additional set of barriers can modulate drug accessibility and bioavailability, often leading to distinct IC_50_ values between parasite stages [37].

Moreover, in the intracellular environment, some compounds may exert dual activity both by acting directly on the amastigotes and by modulating macrophage effector functions. For instance, certain natural and synthetic molecules are known to stimulate macrophage activation pathways, leading to increased production of microbicidal mediators such as nitric oxide and reactive oxygen species (ROS), which contribute to parasite killing [38]. These immunomodulatory effects may enhance antiparasitic activity against amastigotes, explaining cases where IC_50_ values are lower than those observed for extracellular promastigotes.

Thus, *P. peltatum* demonstrated antileishmanial ability, as well as other species of *Piper* that revealed the presence of compounds with important biological activities, such as the antileishmanial activity [39, 40, 41, 42]. As *L. infantum* causes the most severe and potentially fatal clinical manifestations in humans, and given the potent inhibitory actions of HE and 4‐NC (**1**) on promastigotes, we conducted morphological analyses to investigate the cellular changes triggered by these treatments.

**FIGURE 2:**
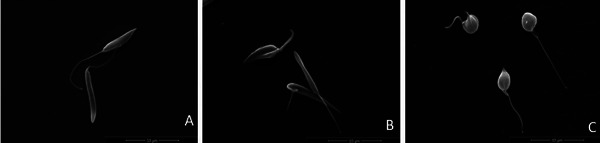
Morphological analysis (scanning electron microscopy) of promastigotes of *Leishmania infantum* that were treated with HE of *Piper peltatum* or 4‐NC (**1**) for 72 h at concentration corresponding to IC_50_. (A) Control parasites had a normal elongated body, with a smooth and intact cell surface. (B) Promastigotes that were treated with extract HE of *P. peltatum* had altered flagellum and smaller cell size. (C) Treatment with 4‐NC (**1**) caused the same alterations as in (B) and the additional surface roughness, reduction in cell body size, and a substantial decrease in cell volume. Scale bar: (A) = 10 µm; (B) = 10 µm; (C) = 10 µm.

Scanning electron microscopy (SEM) analysis revealed that untreated promastigotes displayed the expected morphology, characterized by an elongated body, a prominent flagellum, and a smooth and intact cell surface (Figure 2A). In contrast, parasites treated with HE showed noticeable morphological alterations, including flagellum shortening and surface roughening (Figure 2B). Treatment with 4‐NC (**1**) induced even more pronounced morphological changes, such as marked surface roughness, reduction in cell body size, and a substantial decrease in cell volume, indicating a stronger disruptive effect of the isolated compound on parasite structure (Figure 2C). Notably, despite these alterations, plasma membrane integrity remained preserved in both treatments.

**FIGURE 3:**
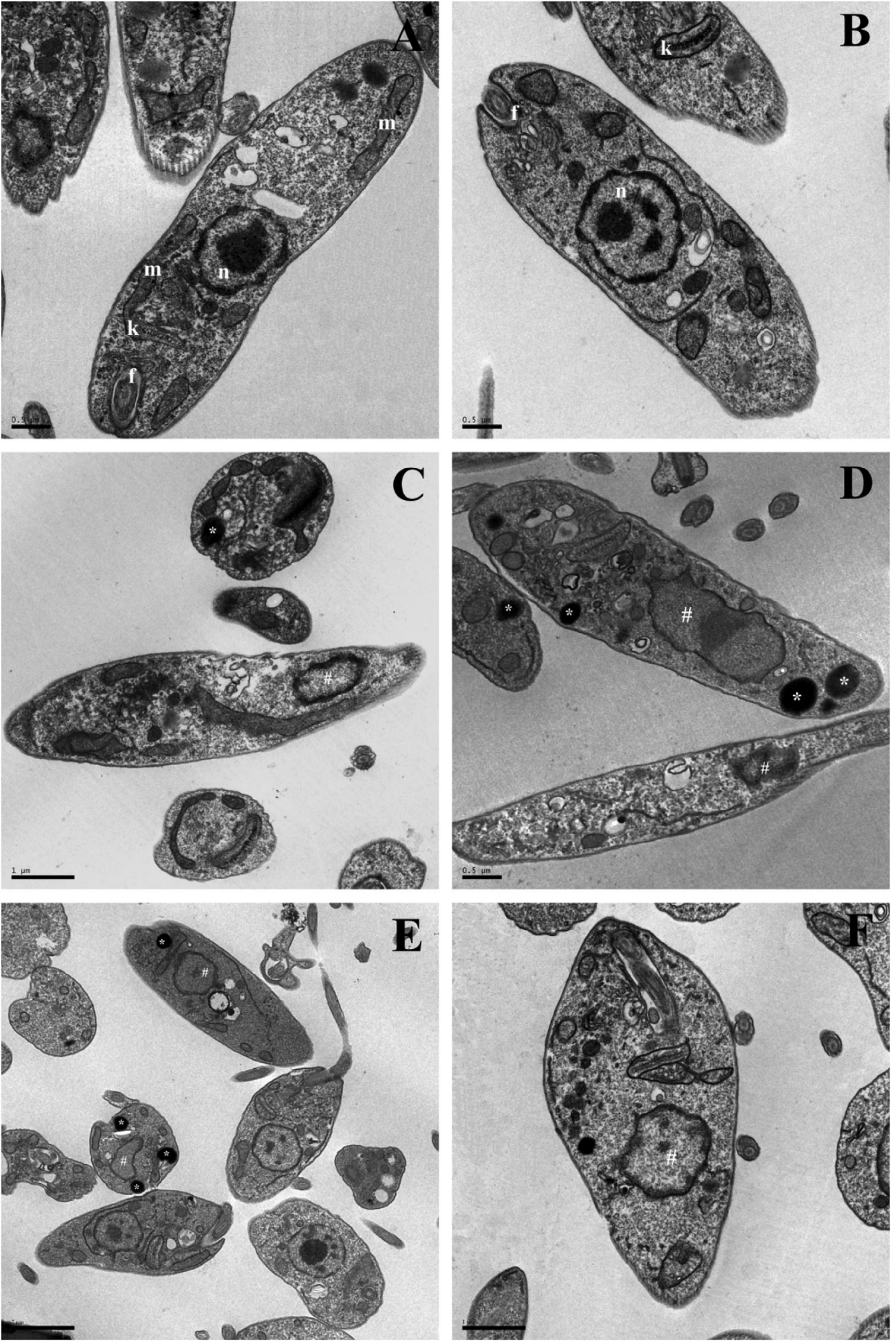
Ultrastructural alterations in promastigote forms of *Leishmania infantum* that were treated with *Piper peltatum* for 72 h at concentrations corresponding to IC_50_. Promastigotes without treatment that presented a normal ultrastructure (A and B); promastigotes treated with HE (C and D); promastigotes treated with 4‐NC (E and F). The asterisks (*) represent lipid‐storage bodies. The # indicates DNA disorganization in nuclei. Scale bar: (A) = 0.9 µm; (B) = 0.5 µm; (C) = 1 µm; (D) = 0.5 µm; (E) = 2 µm; (F) = 1 µm. f, flagellum; k kinetoplast; m, mitochondrion; n nucleus.

**FIGURE 4:**
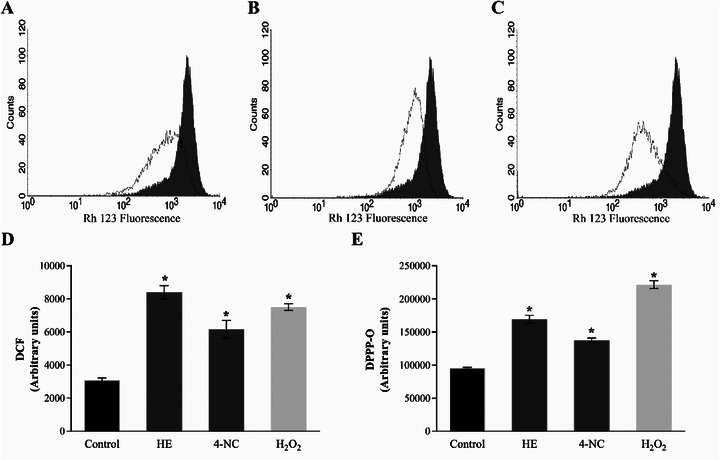
Mitochondrial membrane potential assay in *Leishmania infantum* treated with *Piper peltatum* for 24 h at concentrations corresponding to IC_50_ using Rh123 staining. (A) HE, (B) 4‐NC (**1**), and (C) carbonyl cyanide m‐chlorophenylhydrazone (CCCP), where the gray histogram represents the negative control. (D) Reactive oxygen species production in *L. infantum* treated with HE of *P. peltatum* or 4‐NC (**1**) for 24 h at concentrations corresponding to IC_50_, based on H_2_DCFDA fluorescence. (E) Lipid peroxidation in *L. infantum* treated with HE of *P. peltatum* or 4‐NC (**1**) for 24 h at concentrations corresponding to IC_50_, based on DPPP. Data are expressed as mean ± standard deviation from three independent experiments. Statistical analysis was performed using one‐way ANOVA, with significant differences between means identified by Tukey's post hoc test. Asterisks indicate significant differences relative to the control group (*p* < 0.05). 4‐NC, 4‐nerolidylcatechol; HE, hydroethanolic extract.

Additionally, transmission electron microscopy (TEM) revealed ultrastructural alterations in *L. infantum* promastigotes treated with HE (Figure 3C,D) and 4‐NC (**1**) (Figure 3E,F), including nuclear DNA disorganization and mitochondrial damage. In contrast, untreated control parasites (Figure 3A,B) displayed normal ultrastructure without any observable alterations.

On the basis of these ultrastructural findings, further spectrophotometric assays were conducted to investigate the mechanism of action of HE and 4‐NC (**1**) against *L. infantum* promastigotes. The images showed alterations in the *Leishmania* mitochondria. On the basis of this, we decided to evaluate the Δ*Ψ*_m_ in treated parasites. A marked decrease in Rh123 fluorescence intensity was observed, indicating mitochondrial depolarization in both treatments (Figure 4A–C).

Disruption of Δ*Ψ*_m_ can lead to mitochondrial dysfunctions that compromise parasite viability. One well‐known consequence of mitochondrial depolarization is the increase in ROS production via the electron transport chain [43]. Thus, ROS production was quantified in promastigotes treated with HE and 4‐NC (**1**). Both treatments led to significant increases in ROS levels, exceeding 100% relative to untreated controls (Figure 4D).

Excessive ROS can damage vital cellular components, including lipids, proteins, and nucleic acids [44, 45]. To assess the extent of lipid peroxidation, the DPPP assay was performed (Figure 4E). HE and 4‐NC (**1**) induced lipid peroxidation by 64% and 56%, respectively, compared to control parasites, which indicates substantial damage to the functional and structural integrity of cell membranes [46].

**FIGURE 5:**
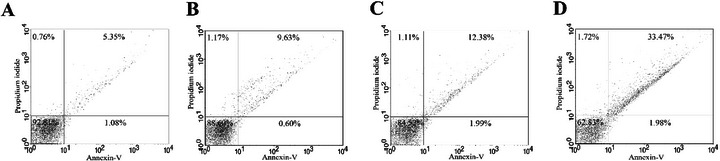
Phosphatidylserine exposure in *Leishmania infantum* treated with *Piper peltatum* for 24 h at concentrations corresponding to IC_50_ using annexin V/FITC and PI. (A) Negative control. (B) HE. (C) 4‐NC (**1**). (D) Actinomycin D. Percentages of annexin V‐positive/PI‐positive cells are shown in the upper and annexin V‐positive/PI‐negative cells are shown in lower right quadrants. Percentages of annexin V‐negative cells are shown in the upper and lower left quadrants.

The cumulative biochemical and morphological alterations suggest a cascade of cellular damage incompatible with parasite survival, prompting further investigation into whether these compounds trigger apoptotic cell death. Apoptosis is characterized by specific biochemical hallmarks such as DNA fragmentation and phosphatidylserine externalization [47]. To assess this, phosphatidylserine exposure was evaluated using annexin V‐FITC/propidium iodide (PI) staining. Promastigotes treated with HE showed 10.23% annexin V‐positivity, whereas those treated with 4‐NC (**1**) showed 14.37%, indicating activation of apoptotic processes (Figure 5).

**FIGURE 6:**
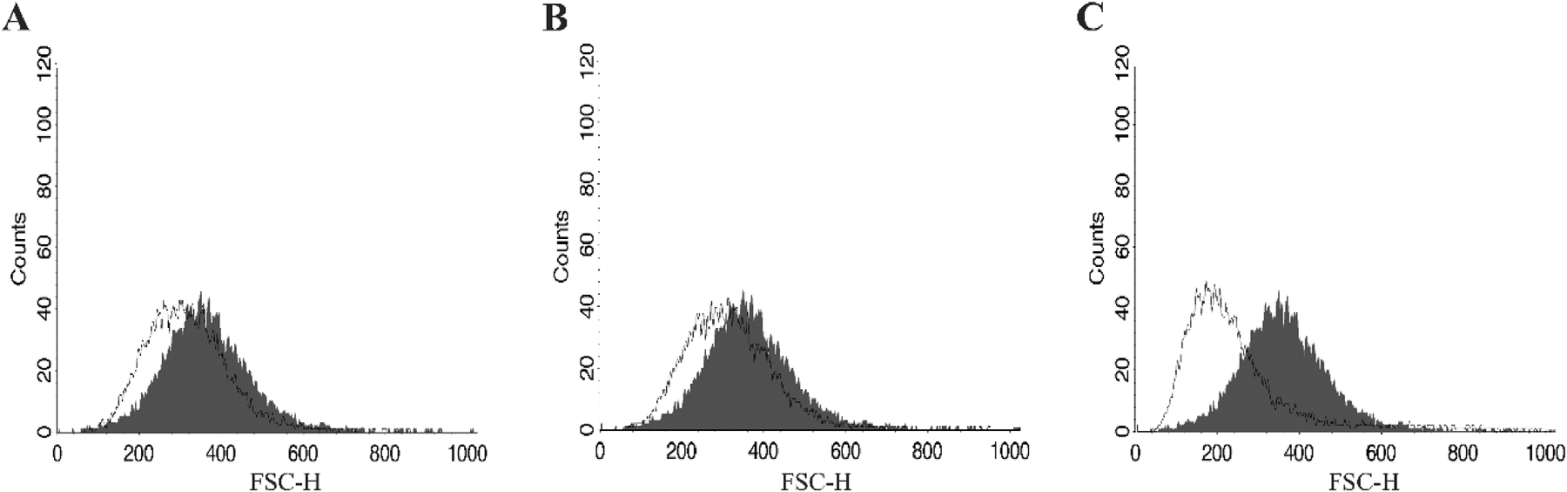
Evaluation of the cell size of *Leishmania infantum* promastigotes treated with *Piper peltatum* for 24 h at concentrations corresponding to IC_50_. (A) HE. (B) 4‐NC (**1**). (C) Actinomycin D. The gray peak represents the negative control.

**FIGURE 7:**
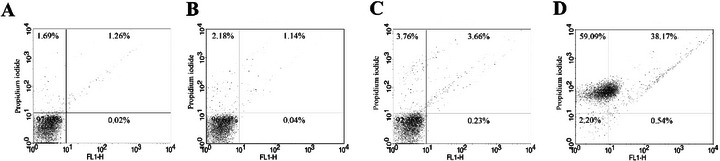
Cell membrane integrity assay in *Leishmania infantum* promastigotes treated with *Piper peltatum* for 24 h at concentrations corresponding to IC_50_ using PI. (A) Negative control. (B) HE. (C) 4‐NC (**1**). (D) Actinomycin D. The percentages of PI‐positive cells are shown in the upper right and left quadrants. The percentages of PI‐negative cells are shown in the lower right and left quadrants.

Reduction in cell volume, another feature of apoptotic death, was observed in parasites treated with both HE and 4‐NC (**1**), though not as pronounced as in the positive control (Figure 6). To further confirm membrane integrity, parasites were stained with PI. No alterations in membrane permeability were observed, supporting the notion that plasma membrane disruption was not a primary event in these treatments (Figure 7).

**FIGURE 8:**
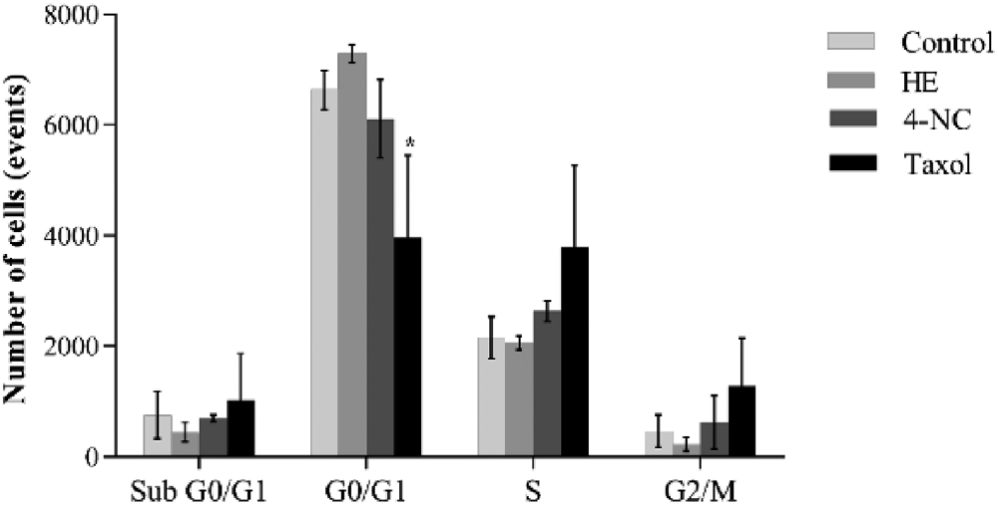
Cell cycle of *Leishmania infantum* treated with HE of *Piper peltatum* or 4‐NC (**1**) for 24 h at concentrations corresponding to IC_50_ values. Data are expressed as mean ± standard deviation from three independent experiments. Statistical analysis was performed using one‐way ANOVA, with significant differences between means identified by Tukey's post hoc test. Asterisks indicate significant differences relative to the control group (*p* < 0.05). 4‐NC, 4‐nerolidylcatechol; HE, hydroethanolic extract.

Cell cycle analysis showed no significant changes in treated parasites, suggesting that parasite death occurred independently of cell cycle arrest (Figure 8). Although autophagy has been proposed as a *Leishmania* cell death mechanism, which is induced by elevated ROS levels, this pathway was not evaluated in the present study [48].

Taken together, the biochemical and morphological alterations induced by HE and 4‐NC (**1**) are consistent with induction of an apoptosis‐like cell death in *Leishmania*. We observed mitochondrial membrane depolarization, a substantial increase in ROS, enhanced lipid peroxidation, phosphatidylserine externalization detectable by annexin V binding, reduction in parasite cell volume, and ultrastructural disruption of mitochondria and nuclear material. These features have been widely reported as hallmarks of apoptosis‐like death in trypanosomatids and are commonly used as convergent indicators when canonical caspase cascades are absent or not clearly conserved in these protozoa [49]. In *Leishmania*, for example, mitochondrial dysfunction and ROS overproduction are frequently upstream events that precipitate chromatin changes and membrane modifications consistent with apoptosis‐like phenotypes [50]. Annexin V binding and cell shrinkage have been similarly interpreted as indicators of membrane and volume alterations associated with apoptotic phenotypes in multiple antileishmanial studies [51, 52]. Notably, several recent reports emphasize that parasite cell death is often apoptosis‐like rather than strictly canonical, and that a combination of markers (mitochondrial depolarization, ROS, lipid peroxidation, annexin V, and ultrastructural evidence) provides a robust indication of programmed cell death in *Leishmania* [53, 54]. Therefore, although our data strongly support an apoptosis‐like mechanism for HE and 4‐NC (**1**) isolated from *P. peltatum*, we acknowledge that definitive molecular proof (e.g., demonstration of metacaspase activation, parasite‐specific proteolytic cleavage products, or DNA laddering) is still required and will be pursued in future work.

## 3 Conclusions

This study reports, for the first time, the antileishmanial activity of the HE, its fractions, and the isolated compound 4‐NC (**1**) from *P. peltatum* leaves against *L. infantum*, *L. amazonensis*, and *L. braziliensis*. HE exhibited the strongest activity, particularly against *L. infantum*, whereas 4‐NC (**1**), despite showing lower potency, demonstrated higher selectivity and reduced cytotoxicity. Fractionation did not improve activity, although some fractions retained moderate efficacy. Morphological and ultrastructural analyses revealed that HE and 4‐NC (**1**) induce mitochondrial dysfunction, ROS overproduction, lipid peroxidation, and phosphatidylserine exposure, leading to parasite death via apoptosis‐like process. These results support the traditional use of *Piper* species and highlight *P. peltatum* as a promising source of bioactive compounds. Given the limitations of current leishmaniasis treatments, our findings contribute to the search for safer and more effective alternatives, warranting further in vivo and mechanistic studies.

## 4 Experimental Section

### 4.1 General Procedures

^1^H and ^13^C NMR spectra were recorded on Varian Mercury Plus spectrometers operating at 300.00 MHz for ^1^H and 75.45 MHz for ^13^C. Spectra were also acquired using DEPT techniques and two‐dimensional correlations from COSY, gHMBC, and gHSQC contour maps. Chemical shifts were reported in ppm relative to TMS as an internal standard. CDCl_3_ was used as the solvent.

Column chromatography was performed using silica gel 60, and filtrations were conducted on Sephadex LH‐20. Column diameters were adjusted according to the mass of the material. Thin layer chromatography (TLC) was performed on normal phase pre‐coated silica gel 60G or 60GF254 plates (Merck) with a 0.25 mm thickness. Compounds were visualized by UV irradiation at 254 and 366 nm and/or by spraying with an H_2_SO_4_/anisaldehyde/acetic acid solution (1:0.5:50 mL) followed by heating at 100°C.

### 4.2 Plant Material

*P. peltatum* leaves were collected in April 2012 at Florentino Farm, in Novo Progresso, Pará (7°06′43.82″ S 55°23′42.54″ W). A voucher specimen was deposited in the Tangará Herbarium, Mato Grosso State University (TANG 1777) by professor Diones Krinski. The plant was weighed and dried in air circulating oven at 36°C, grounded in a knife mill, and packed and stored in a dry place away from light.

### 4.3 Extraction and Isolation

The dried and ground leaves (700 g) were extracted with cold ethanol/water (9:1). After evaporation under reduced pressure, an HE was obtained (26.5 g), which was subjected to vacuum column chromatography (silica gel, 150 g) and eluted sequentially with HF (1000 mL), hexane/dichloromethane 1:1 v/v (1200 mL) (HDF), DF (1000 mL), dichloromethane/ethyl acetate 1:1 v/v (1000 mL) (DEAF), EAF (700 mL), and MF (700 mL). Fractions were analyzed by TLC and ^1^H NMR.

DEAF (1.0 g), HF (2.0 g), and HDF (2.0 g) fractions were further fractionated by column chromatography on silica gel 60 (70–230 mesh), eluted with gradients of hexane, hexane/dichloromethane (49:1, 19:1, 9:1, and 1:1 v/v), dichloromethane, dichloromethane/ethyl acetate (49:1, 19:1, 9:1, and 1:1 v/v), ethyl acetate, and methanol.

A total of 71 fractions of the DEAF were obtained, from which DEAF 30 resulted in the compound (**1**) (12.5 mg). The HF yielded 110 fractions, with the fractions HF 40–54 (128 mg) pooled and fractionated on Sephadex LH 20 silica using chloroform/methanol 1:1 v/v as the mobile phase, providing the compound (**1**) (25.0 mg). The HDF provided 100 fractions, whereas the HDF 32–44 and HDF 56–60 fractions were pooled (70.0 and 56.0 mg, respectively) and fractionated on Sephadex LH 20 silica using chloroform/methanol 1:1 v/v as the mobile phase provided compound (**1**) (17.0 mg).

4‐NC (**1**): ^1^H NMR (300 MHz‐CDCl_3_) *δ*: 6.85 (d, *J *= 2.1 Hz, H2;1H); 6.79 (d, *J *= 8.1 Hz, H6; 1H); 6.75 (dd, *J *= 8.4, 2.1 Hz, H6; 1H); 5.99 (dd, *J *= 17.4, 10.8 Hz, H2′; 1H); 5.11–5.12 (m, H10′; 4H); 5.07 (dd, *J *= 10.2, 2.1 Hz, H1′; 1H); 5.02 (dd, *J *= 17.1, 2.1 Hz, HB; 1H); 2.01–2.06 (m, H9′; 4H); 1.92–1.97 (m, H8′; 4H); 1.81–1.85 (m, H5′, 4H); 1.70–1.79 (m, H4′; 4H); 1.68 (d, *J *= 0.9 Hz, H14′; 1H); 1.60 (d, *J *= 0.6 Hz, H15′; 1H); 1.52 (d, *J *= 1.2 Hz, H13′; 1H); 1.33 (s, H12′; 1H). ^13^C NMR (75 MHz‐CDCl_3_) *δ*: 15.9 (C13′); 17.5 (C15′); 24.3 (C5′); 25.6 (C12′); 25.9 (C14′); 27.7 (C9′); 40.8 (C8′); 42.5 (C4); 44.7 (C3′); 111.4 (C1′); 115.24 (C2); 115.8 (C5); 119 (C6); 125.4 (C10′); 126 (C6′); 132 (C11′); 135.6 (C7′); 140.2 (C1); 144.2 (C4); 145.8 (C3); 149 (C2′).

### 4.4 Biological Assays

#### 4.4.1 Parasites and Macrophages

Experiments were performed using the *L. amazonensis* (MHOM/BR/77/LTB0016), *L. braziliensis* (MCAN/BR/98/R619), and *L. infantum* (MHOM/MA/67/ITNAB263). Parasites were maintained at 26°C, in Schneider medium (Sigma, Saint Louis, EUA) supplemented with 10% fetal bovine serum (FBS), 100 IU mL^−1^ penicillin, and 100 µg mL^−1^ streptomycin.

Macrophages were obtained by peritoneal lavage of Swiss mice using cold RPMI 1640 medium, supplemented with 10% FBS, and incubated at 37°C under 5% CO_2_. Animal experiments were approved by the Ethical Committee on Animal Use (CEUA) of the State University of Maringá (Protocol 9749241017).

#### 4.4.2 In Vitro Anti‐Promastigote Activity

The evaluation of anti‐promastigote activity was performed in accordance with Denizot and Lang [55], with adaptations. It evaluated the anti‐promastigote activity of the HE, HF, HDF, DF, DEAF, EAF, MF, and 4‐NC (**1**) from *P. peltatum*. Dimethylsulfoxide (DMSO) was used to solubilize the samples, and the final DMSO concentration did not exceed 0.1%. Serial dilutions of samples were added to promastigotes cultures of *L. amazonensis*, *L. braziliensis*, and *L. infantum* and incubated for 72 h at 26°C. These assays were performed in triplicate using a 96‐well flat bottom plate (FalconCo, Franklin Lakes, USA). Leishmanicidal activity was assessed using the resazurin microtiter assay plate (REMA) method and was quantified in a Spectra Max GEMINI XPS (Molecular Devices, Silicon Valley, USA) spectrofluorometer with excitation at 560 nm and emission at 590 nm. The calculation of IC_50_ (inhibition concentration for 50% of parasites growth) was determined by logarithmic regression analysis in GraphPad Prism 8.0.

#### 4.4.3 In Vitro Intracellular Anti‐Amastigote Activity

To evaluate activity against intracellular amastigotes, the macrophages obtained from peritoneal lavage were adjusted to a concentration of 1 × 10^6^ macrophages mL^−1^ and plated onto LAB‐TEK chambers (Nunc, NY, USA). After 1 h, the cultures were washed to remove non‐adherent cells. The remaining cells were incubated at 37°C, 5% CO_2_, with promastigotes of *L. amazonensis* or *L. braziliensis* or *L. infantum* at a ratio of 3:1. After 4 h, the chambers were washed to remove non‐internalized parasites. The samples were incubated at different concentrations of 0–50 µg mL^−1^ for 72 h at 37°C and 5% CO_2_. Following the incubation period, the activity was evaluated microscopically by dying the chambers with Instant Prov (Newprov, Curitiba, Brazil). On a light microscope, 100 macrophages per chamber were evaluated to determine the number of macrophages infected and the number of amastigotes within each infected macrophage. The results were expressed as infection rate (IR): IR = (% infected cells × amastigote average per infected macrophage). IR of amastigotes from untreated infected macrophages (negative control—NC) was considered 100% of survival for the purpose of IC_50_ calculation. The IC_50_ calculation was carried out by logarithmic regression using GraphPad Prism 8.0.

#### 4.4.4 Cytotoxicity Assay

The cytotoxicity of the samples was evaluated fluorometrically with resazurin. Initially, macrophages obtained from peritoneal Swiss mice were plated (1 × 10^6^ cells mL^−1^) in RPMI 1640 medium supplemented with 10% of FBS and incubated for 1 h at 37°C with 5% CO_2_. The culture was washed, and samples were added in different concentrations (0–400 µg mL^−1^) for 72 h at 37°C. As a positive control, macrophages were incubated with 0.1% Triton X‐100 for complete lysis. After the incubation period, the supernatant was removed, and 200 µL of PBS containing 22 µL of resazurin was added. Following a 3 h incubation period, viability was measured with a spectrofluorometer (Spectra Max GEMINI XPS‐Molecular Devices, Silicon Valley, USA) with excitation at 560 nm and emission at 590 nm. The cytotoxic concentration for 50% of the cells (CC_50_) was determined in relation to the control by logarithmic regression analysis using GraphPad Prism 8.0. The SI was calculated by the CC_50_/IC_50_ formula on intracellular amastigotes.

#### 4.4.5 Morphological and Ultrastructural Analyses

To evaluate the morphological and ultrastructural changes on promastigotes of *L. infantum* that were induced by the HE of *P. peltatum* and 4‐NC (**1**), SEM and TEM were performed. For that, promastigotes of *L. infantum* were treated with HE of *P. peltatum* or 4 NC (**1**) with the value corresponding to IC_50_ for 72 h. After treatment, parasites were washed with PBS and fixed in 2.5% sodium glutaraldehyde in 0.1% sodium cacodylate buffer at 4°C overnight. For TEM, promastigotes were postfixed with 1% osmium tetroxide (OsO_4_), 0.8% ferrocyanide of potassium, and 10 mM CaCl_2_ in 0.1 M cacodylate buffer, dehydrated in increasing acetone gradient, and soaked in EPON resin for 72 h at 60°C. Ultrafine sections were obtained, stained with uranyl acetate and lead citrate, and examined using a transmission electron microscope JEM 1400 (JEOL). For SEM analysis, promastigotes were placed on a glass sample holder coated with poly‐l‐lysine, dehydrated through a graded ethanol series, critical‐point dried in CO_2_, gold plated, and examined using a Quanta 250 scanning electron microscope (FEI).

#### 4.4.6 Effect of HE and 4‐NC (1) on Mitochondrial Membrane Potential (Δψm)

Promastigotes of *L. infantum* were incubated with HE extract of *P. peltatum* IC_50_ or 4‐NC (**1**) IC_50_ for 24 h. After incubation, the cells were collected by centrifugation, washed in PBS, and incubated with an Rh123 solution (5 mg mL^−1^ in ethanol) for 15 min. Then, the cells were washed again, resuspended in PBS, and incubated for an additional 30 min. Next, 10 000 events were acquired using a FACSCalibur flow cytometer equipped with CellQuest software for data analysis. CCCP (100 µM) was used as a positive control [56].

#### 4.4.7 Detection of ROS

To evaluate the production of ROS, the promastigotes were treated with HE or 4‐NC (**1**) with 5.6 and 17.4 µg mL^−1^, respectively (IC_50_), for 24 h, then washed and loaded with 10 µM H_2_DCFDA for 45 min without light. The fluorescence was determined in a Victor X3 spectrofluorometer at *λ*_ex_ = 488 nm and *λ*_em_ = 530 nm. Hydrogen peroxide (H_2_O_2_) (20 mM) was used as positive control [57].

#### 4.4.8 Determination of Lipid Peroxidation

Lipid peroxidation was assessed using the DPPP (diphenyl‐1‐pyrenylphosphine) probe. The promastigotes were treated with HE or 4‐NC (**1**) with 5.6 and 17.4 µg mL^−1^, respectively, for 24 h and compared with the substances above. The fluorescence intensities of samples were measured with a fluorescence microplate reader (Victor X3, PerkinElmer, Finland), at *λ*_ex_ = 355 nm and *λ*_em_ = 460 nm. The results were compared with 20 mM hydrogen peroxide (positive control) [58].

#### 4.4.9 Phosphatidylserine Exposure

The phosphatidylserine exposure was determined by annexin V‐FITC/PI staining. Promastigotes were treated with HE or 4‐NC (**1**) with 5.6 and 17.4 µg mL^−1^, respectively, for 24 h. After incubation, cells were washed and resuspended in 100 µL of binding buffer (140 mM NaCl, 5 mM CaCl_2_, and 10 mM HEPES‐Na, pH 7.4), followed by the addition of 5 µL of annexin V‐FITC, for 15 min. Binding buffer (400 µL) and 50 µL of PI were then added. Antimycin A (125.0 µM) was used as a positive control. Data acquisition (10 000 events) and analysis were performed using a FACSCalibur flow cytometer equipped with Cell Quest software. Cells stained with annexin V (PI positive or negative) were considered apoptotic, and cells that were positive only for PI were considered necrotic [59].

#### 4.4.10 Determination of Cell Size

To evaluate changes on cell volume, promastigotes were treated with HE or 4 NC (**1**) with IC_50_ values for 24 h, washed with PBS, and analyzed using fluorescence‐activated cell sorting on a FACSCalibur flow cytometer. Actinomycin D (20.0 mM) was used as positive control. The results were analyzed using CellQuest software, and forward light scatter (FSC‐H) was used as an indicator of cell size [60].

#### 4.4.11 Determination of Cellular Membrane Integrity

The promastigotes were treated with the IC_50_ of HE or 4‐NC (**1**), 5.6 and 17.4 µg mL^−1^, respectively, for 24 h, washed with PBS, and incubated with 10 µg mL^−1^ PI for 10 min. Digitonin (40.0 µM) was used as positive control. After that, promastigotes were analyzed using FACSCalibur flow cytometer equipped with CellQuest software, at total of 10 000 events. Alteration in fluorescence of PI was quantified in percentage and compared with positive control [60].

#### 4.4.12 Cell Cycle Analysis

In order to evaluate possible changes in the cell cycle, promastigotes were treated with HE or 4‐NC (**1**) with 5.6 and 17.4 µg mL^−1^, respectively, for 24 h and fixed in 70% cold methanol–PBS at 4°C for 1 h. After that, cells were washed in PBS and 10 µL of PI (10 µg mL^−1^). Then, 10 µL RNAse A (10 µg mL^−1^) was added, and the cells were incubated at 37°C for 45 min. The data acquisition and analysis were performed using a FACSCalibur flow cytometer equipped with Cell Quest software. The percentages of cells in stage of cell cycle were determined, and a total of 10 000 events were acquired [58].

### 4.5 Statistical Analysis

Data are expressed as mean ± standard deviation from at least three independent experiments. Statistical analysis was performed using one‐way analysis of variance (ANOVA), with significant differences between means identified by Tukey's post hoc test. Values of *p* < 0.05 were considered statistically significant. The analyses were carried out using the GraphPad Prism 8.0 software.

## Author Contributions

**Fabiana Brusco Lorenzetti**: writing – original draft, investigation, methodology, data curation, visualization, validation, formal analysis. **Rodolfo Bento Balbinot**: writing – review and editing, methodology, data curation, visualization. **Danielle Lazarin‐Bidóia**: writing – original draft, methodology, formal analysis. **Viviane dos Santos Faiões**: methodology, formal analysis. **Eduardo Caio Torres‐Santos**: methodology, formal analysis. **Celso Vataru Nakamura**: supervision, resource, validation. **Tânia Ueda‐Nakamura**: supervision, writing – review and editing. **Diógenes Aparício Garcia Cortez**: conceptualization, supervision. **Benedito Prado Dias Filho**: writing – review and editing, conceptualization, supervision, resource, validation.

## Conflicts of Interest

The authors declare no conflicts of interest.

## Supporting information




**Supporting File 1**: cbdv70881‐sup‐0001‐SuppMat

## Data Availability

The data that support the findings of this study are available in the supporting information of this article.
