# Supplementary material for: Unveiling the Leishmanicidal Mechanism of 4‐Nerolidylcatechol Isolated From Piper peltatum Against Leishmania infantum
Source: Chem Biodivers. 2026 Jan 10;23(1):e03244. doi: 10.1002/cbdv.202503244 (PMC12790417; doi:10.1002/cbdv.202503244)
Supplement: Supplementary file 1 — Supporting File 1: cbdv70881‐sup‐0001‐SuppMat [file CBDV-23-e03244-s001.pdf]

Structural Determination of 4-Nerolidylcatechol Isolated from the Leaves of *Piper peltatum L*.

The compound 4-NC (Figure 1), with molecular formula $C_{21}H_{30}O_2$, was first described by Anake Kijjoa in 1980, who isolated the substance from the hexane fraction of the root and leaf extract of *Piper peltatum*. For the identification of the compound, the ^{1}H NMR spectrum was analyzed, revealing an aromatic and an aliphatic portion, referred to as catechol and nerolidyl, respectively.

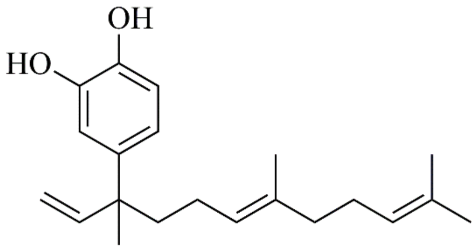

(1)

Figure 1 Chemical structure of 4-nerolidylcatechol.

The aromatic catechol portion was identified by the chemical shifts and coupling constants of three aromatic hydrogens at δ 6.85 (d, 2.1 Hz), δ 6.75 (dd, 4.4 and 2.1 Hz), and δ 6.79 (d, 8.1 Hz). The aliphatic nerolidyl portion was evidenced by the presence of two methyl groups attached to a non-protonated sp^2 carbon, as well as one methyl group and a fully substituted vinyl terminus bound to an sp^3 carbon, at δ 1.52 (d, 1.2 Hz; CH_3-13′), δ 1.68 (d, 0.9 Hz; CH_3-14′), and δ 1.60 (d, 0.6 Hz; CH_3-15′), respectively.

The ^{1}H NMR spectrum also showed a catechol substituted at C-4. The higher chemical shift of this carbon relative to unsubstituted catechol indicated the presence of a substituent, either a tertiary or quaternary carbon (Table 1).

In the proton spectrum, a methyl group was observed at C-12′ (δ 1.33, s), along with two doublets for the olefinic carbon C-1′ at δ_H-A 5.07 (dd, 10.2 and 2.1 Hz) and δ_H-B 5.02 (dd, 17.1 and 2.1 Hz).

The remaining signals correspond to an aliphatic chain whose chemical shifts are almost identical to those of nerolidol, except for the region near the non-protonated sp³ carbon, now bound to carbon rather than oxygen.

In addition to the literature data acquired using 270 MHz (^{1}H NMR) and 60 MHz (^{13}C NMR), the ^{1}H NMR spectrum (Figure 6) reveals two characteristic portions: the aliphatic chains and the aromatic part. In proton nuclear magnetic resonance, the electronic density around a hydrogen atom influences its magnetic shielding effect. Lower electron density results in decreased shielding and consequently lower-field (downfield) chemical shifts. Increased shielding corresponds to higher energy required for resonance, meaning higher frequency and magnetic field.

The methyl carbons appear in the aliphatic region at higher-field signals: H-12′ at δ_H 1.33 ppm (s); H-13′ at δ_H 1.52 ppm (d, J = 1.2 Hz); H-14′ at δ_H 1.68 ppm (d, J = 0.9 Hz); and H-15′ at δ_H 1.60 ppm (d, J = 0.6 Hz). H-2′, H-14′ and H-15′ couple with C-12′, and the methyl groups couple with one another. The methylene carbons absorb at higher field, except for the hydrogens at C-1′, where the sp^2 environment results in downfield signals: H-A at δ_H 5.07 ppm (dd, J = 10.2 and 2.1 Hz) and H-B at δ_H 5.02 ppm (dd, J = 17.1 and 2.1 Hz). Additional methylene protons appear at H-5′: δ_H 1.81–1.85 ppm (m); H-4′: δ_H 1.70–1.77 ppm (m); H-8′: δ_H 1.92–1.97 ppm (m); and H-9′: δ_H 2.01–2.06 ppm (m). H-12′ couples with C-4′ over long range; H-8′ and H-9′ couple with each other; and C-13′ couples with H-8′. These correlations were confirmed by HMBC and HSQC spectra (Figures 7 and 8).

The ^{1}H–^{13}C HMBC spectrum (^{2}J_CH, ^{3}J_CH) enabled further assignments. The ^{1}H NMR spectrum of 4-NC exhibits aromatic methine signals at H-2: δ_H 6.85 ppm (d, J = 2.1 Hz); H-6: δ_H 6.75 ppm (dd, J = 8.4 and 2.1 Hz); and H-5: δ_H 6.79 ppm (d, J = 8.1 Hz). H-2 couples with C-6 and H-6 couples with C-2 at meta positions. Other methine carbons include H-2′ at δ_H 5.99 ppm (dd, J = 17.4 and 10.8 Hz); H-6′ at δ_H 5.11–5.12 ppm (m); and H-10′ at δ_H 5.11–5.12 ppm (m). H-12′, H-A, and H-B couple with C′-H at short range. H-5′ and H-13′ couple with C-6′, and the hydrogens have the same chemical shift as H-10′ due to chemical equivalence. H-9′, H-14′, and H-15′ couple with C-11′.

A proton signal may be split by a neighboring proton if they occupy different chemical environments. If hydrogens HA and HB are not chemically equivalent, the HA signal will be split by HB, appearing as a doublet. Thus, HA and HB (on adjacent carbons) couple with each other, and the coupling constant (J) is measured in hertz (Hz). If two HB hydrogens were present, the HA signal would appear as a triplet, with the central peak twice the height of each outer peak

Table 1: Long-range hydrogen–carbon interactions (^{2}JCH, ^{3}JCH) observed in the two-dimensional heteronuclear correlation spectra ^{1}H–^{13}C HSQC (300 MHz) and ^{1}H–^{13}C HMBC (300 MHz), in $CDCl_3$ of the compound 4-Nerolidylcatechol. Chemical shifts (δH, ppm) from the literature (Kijjoa, 1980) and experimental data."

Carbon	δ_H (KIJJOA,1980)	^{1}Hx ^{13}C-HSQC δ_H (experimental)	^{1}Hx ^{13}C-HMBC $^2J_{CH}$	$^3J_{CH}$
C-4			H-6	H-3
C-3				H-5
CH-2	6.83(d,j=2.0 Hz)	6.85 (*d*, 2.1)		H-6
C-1				H-12´; H-6
CH-6	6.78 (dd, j=8,0 Hz.	6,75 (*dd*, 8.4; 2.1)		H-2
CH-5	6.73(dd, j=8.0 e 2.0 Hz))	6,79 (*d*, 8.1)		
CH_2-1´	HA-5.05 (dd,j=17.0 e 1.3Hz) HB-5.00 (dd, j=17.0 e 1.3Hz)	HA- 5.07 (*dd*, 10;2; 2.1) HB- 5.02 (*dd*, 17.1; 2.1)		
CH-2´	5.97 (dd, j=17.0 e 10.5 Hz)	5,99 (*dd*, 17.4; 10,8)	H-A; H-B; H-12´	

C-3´			H-2´; H-12´	H-3; H-5; H-A; H-B
CH_2-4´	1.6-1.9 (m, 4H)	1.70- 1.79 (*m*)		H-12´
CH_2-5´	1.9-2.1 (m, 4H)	1.81- 1.85 (*m*)		
CH-6´	5.09 (t; j= 7.5 Hz)	5.11- 5.12 (*m*)	H-5´	H-13´
C-7´			H-13´; H-8´	
CH_2-8´	1.9-2.1 (m, 4H)	1.92-1.97 (*m*)	H-9´	H-13´
CH_2-9´	1.9-2.1 (m, 4H)	2.01-2.06 (*m*)	H-8´	
CH-10´	5.09 (t; j=7.5Hz)	5.11- 5.12 (*m*)	H-9´	H-14´; H-15´
C-11´			H-15´; H-14´	H-9´
CH_3-12´	1.30 (s)	1.33 (*s*)		H-2´
CH_3-13´	1.67 (s)	1.52 (*d*, 1.2)		H-8´
CH_3-14´	1.59 (s)	1.68 (*d*, 0.9)		H-15´
CH_3-15´	1.51 (s)	1.60 (*d*, 0.6)		H-14´

Table 2: Comparative ^{13}C NMR (75 MHz) chemical shift (δC, ppm) data from the literature (Kijjoa, 1980) and the experimental results in $CDCl_3$ for the compound 4-Nerolidylcatechol

Carbono	δ_H (KIJJOA,1980)	**^{1}Hx ^{13}C-HSQC** δ_H (experimental)	**^{1}Hx ^{13}C-HMBC** $^2J_{CH}$	$^3J_{CH}$
C-4			H-6	H-3
C-3				H-5
CH-2	6.83(d,j=2.0 Hz)	6,85 (*d*, 2.1)		H-6
C-1				H-12´; H-6
CH-6	6.78 (dd, j=8,0 Hz)	6.75 (*dd*, 8.4; 2.1)		H-2
CH-5	6.73(dd, j=8,0 e 2.0 Hz))	6.79 (*d*, 8.1)		
CH_2-1´	HA-5.05 (dd,j=17.0 e 1.3Hz) HB-5.00 (dd, j=17.0 e 1.3Hz)	HA- 5.07 (*dd*, 10.2; 2.1) HB- 5.02 (*dd*, 17.1; 2,1)		
CH-2´	5.97 (dd, j=17.0 e 10.5 Hz)	5.99 (*dd*, 174; 10,8)	H-A; H-B; H-12´	
C-3´			H-2´; H-12´	H-3; H-5; H-A; H-B
CH_2-4´	1.6-1.9 (m, 4H)	1.70- 1.79 (*m*)		H-12´
CH_2-5´	1.9-2.1 (m, 4H)	1.81- 1.85 (*m*)		
CH-6´	5.09 (t; j= 7.5 Hz)	5.11- 5.12 (*m*)	H-5´	H-13´
C-7´			H-13´; H-8´	
CH_2-8´	1.9-2.1 (m, 4H)	1.92-1.97 (*m*)	H-9´	H-13´
CH_2-9´	1.9-2.1	2.01-2.06 (*m*)	H-8´	

	(m, 4H)			
CH-10´	5.09 (t; j=7.5Hz)	5.11- 5.12 (*m*)	H-9´	H-14´; H-15´
C-11´			H-15´; H-14´	H-9´
CH_3-12´	1.30 (s)	1.33 (*s*)		H-2´
CH_3-13´	1.67 (s)	1.52 (*d*, 1.2)		H-8´
CH_3-14´	1.59 (s)	1.68 (*d*, 0.9)		H-15´
CH_3-15´	1.51 (s)	1.60 (*d*, 0.6)		H-14´

The ^{13}C NMR spectrum (Figures 9 and 10) showed the presence of four quaternary carbons, five methylene carbons, six methine carbons, and six methyl carbons.

The multiplicity of these carbons was verified by DEPT analysis (Figure 11), in which the five methylene carbons were highlighted, as they appeared with downward signals, with chemical shifts δC 111.4, 42.5, 24.3, 40.8, and 27.2 ppm, assigned to C-1′, C-4′, C-5′, C-8′, and C-9′.

The signals at δC 144.2 and 145.8 ppm were assigned to C-4 and C-3, respectively, which are bonded to hydroxyl groups.

The chemical shifts δC 25.6, 15.9, 25.9, and 17.5 ppm were attributed to the methyl carbons C-12′, C-13′, C-14′, and C-15′, which appear upfield because they are less deshielded, being attached to an aliphatic carbon chain.

The aromatic methine carbons are C-2, C-5, and C-6, showing δC 115.24, 115.8, and 119.0 ppm, respectively.

The remaining quaternary carbons are not located in the aromatic region and show δC 149.0, 126.0, and 125.4 ppm, corresponding to C-2′, C-6′, and C-10′.

The signals at 140.2, 149.0, 132.0, and 135.0 ppm belong to the quaternary carbons C-1, C-3′, C-11′, and C-7′ (Table 2).

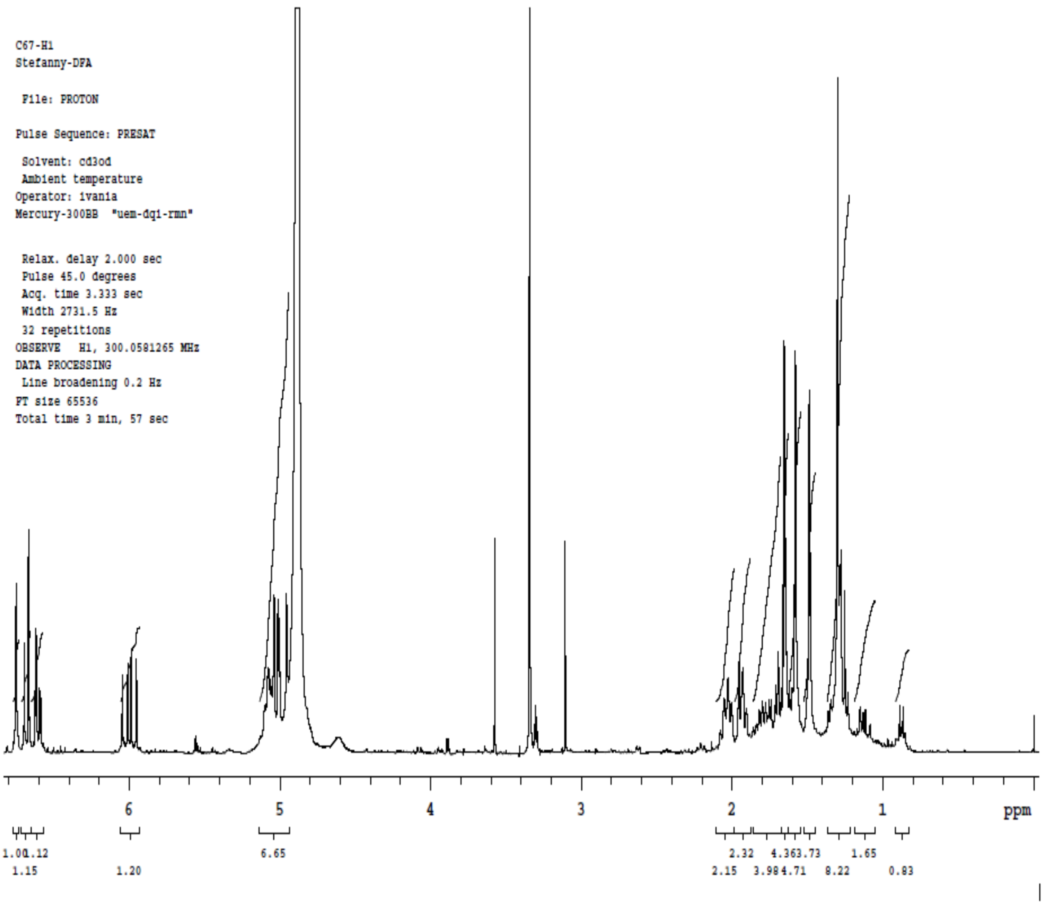

^{1}H NMR spectrum of 4-Nerolidylcatechol (300 MHz, $CDCl_3$)

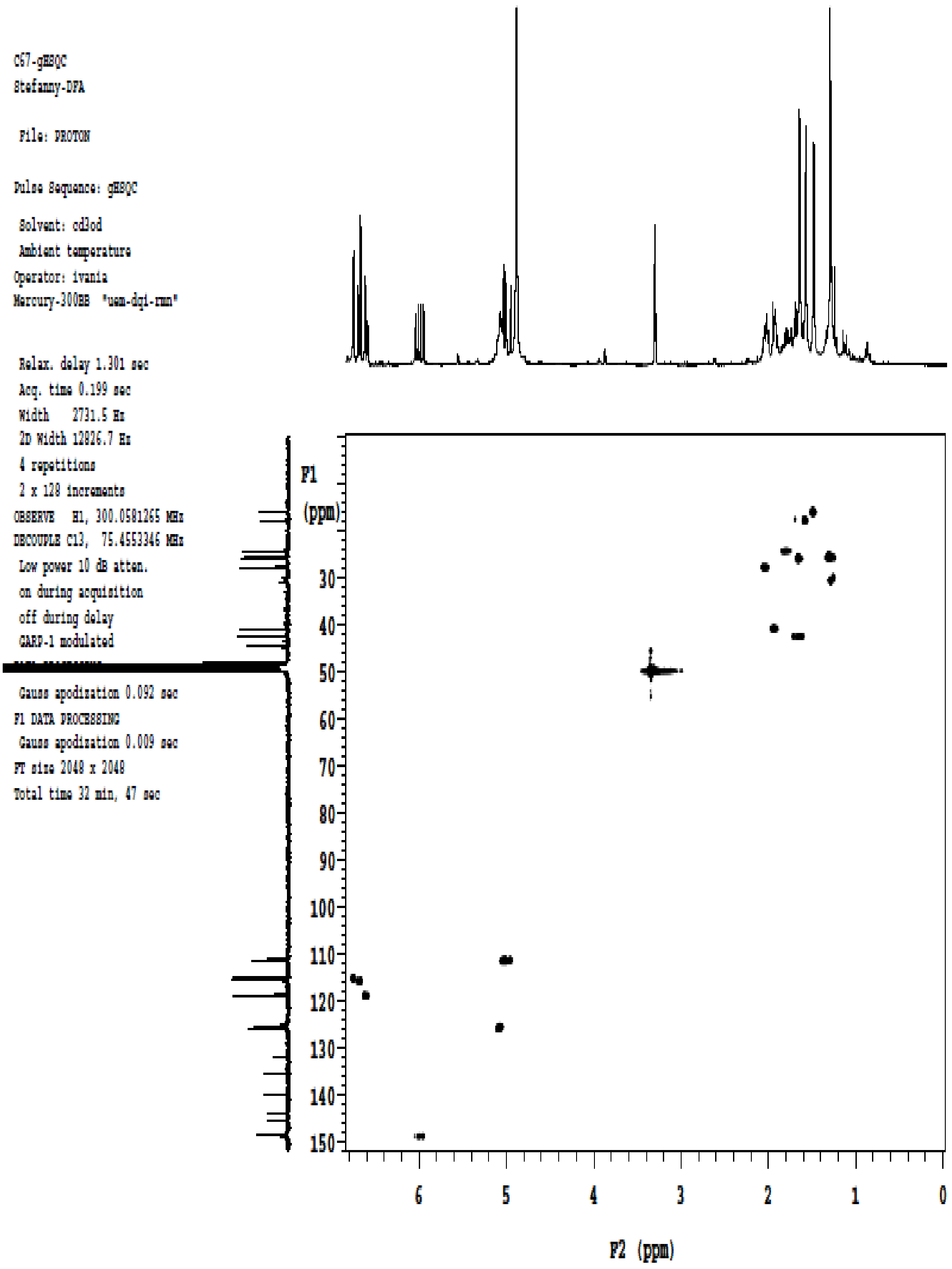

^{1}H–^{13}C HSQC spectrum of 4-nerolidylcatechol (300 MHz, $CDCl_3$)."

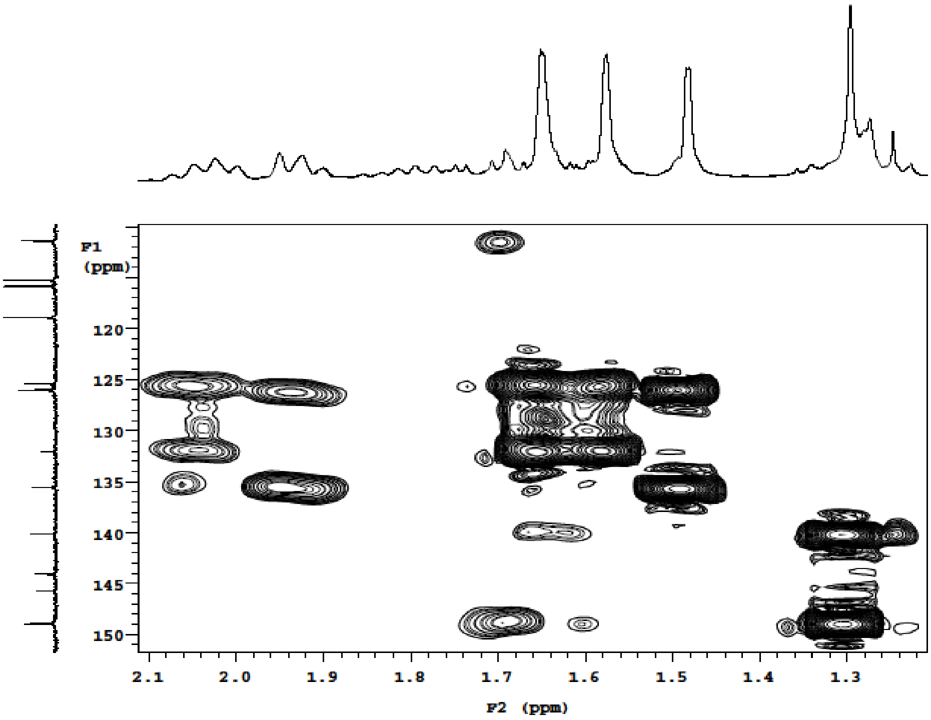

^{1}H–^{13}C HMBC spectra of compound 4-NC (300 MHz, $CDCl_3$).

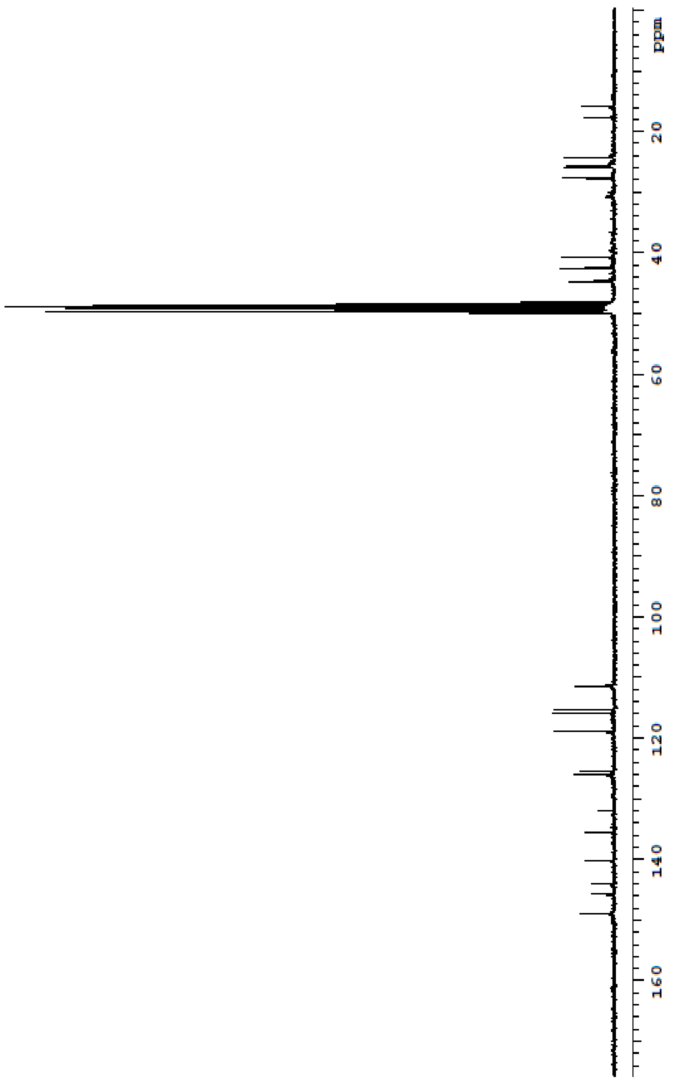

^{13}C NMR spectrum of the compound 4-Nerolidylcatechol (75.45 MHz, $CDCl_3$)

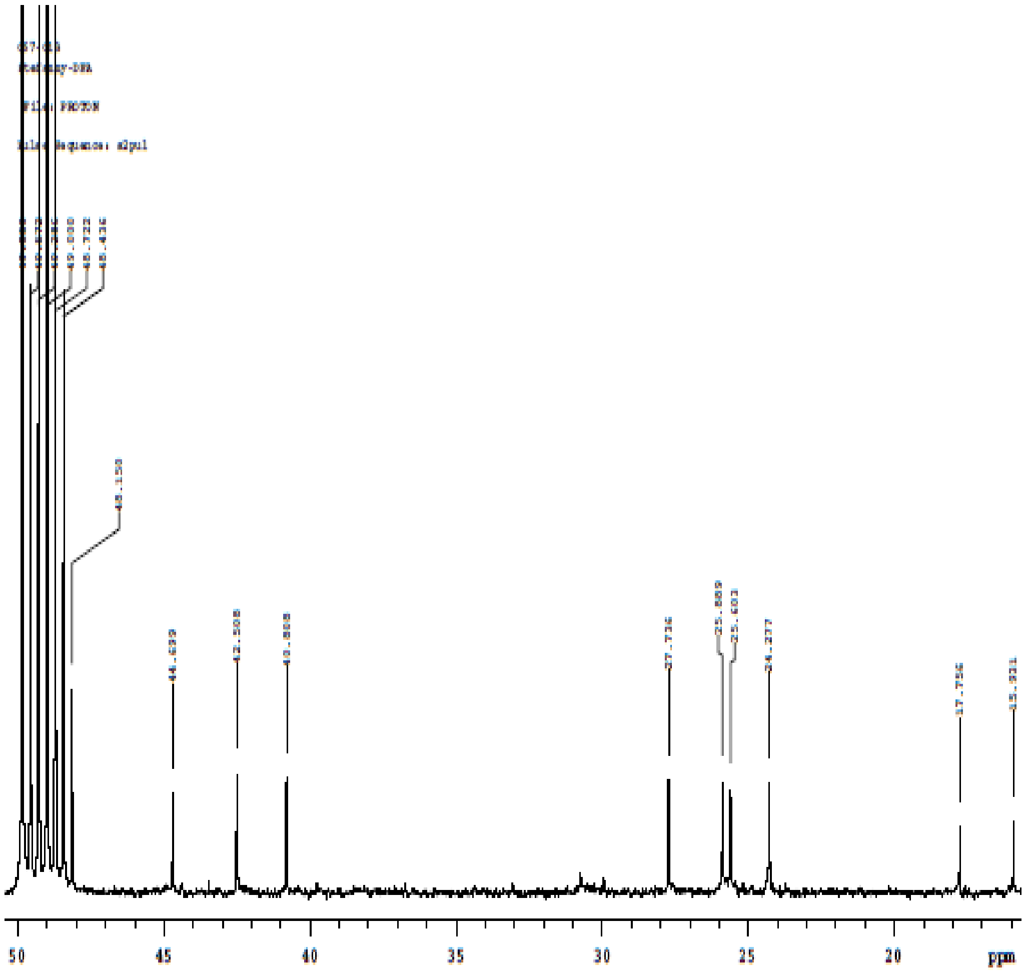

Detailed view of the ^{13}C NMR spectrum of 4-Nerolidylcatechol (75.45 MHz, $CDCl_3$)

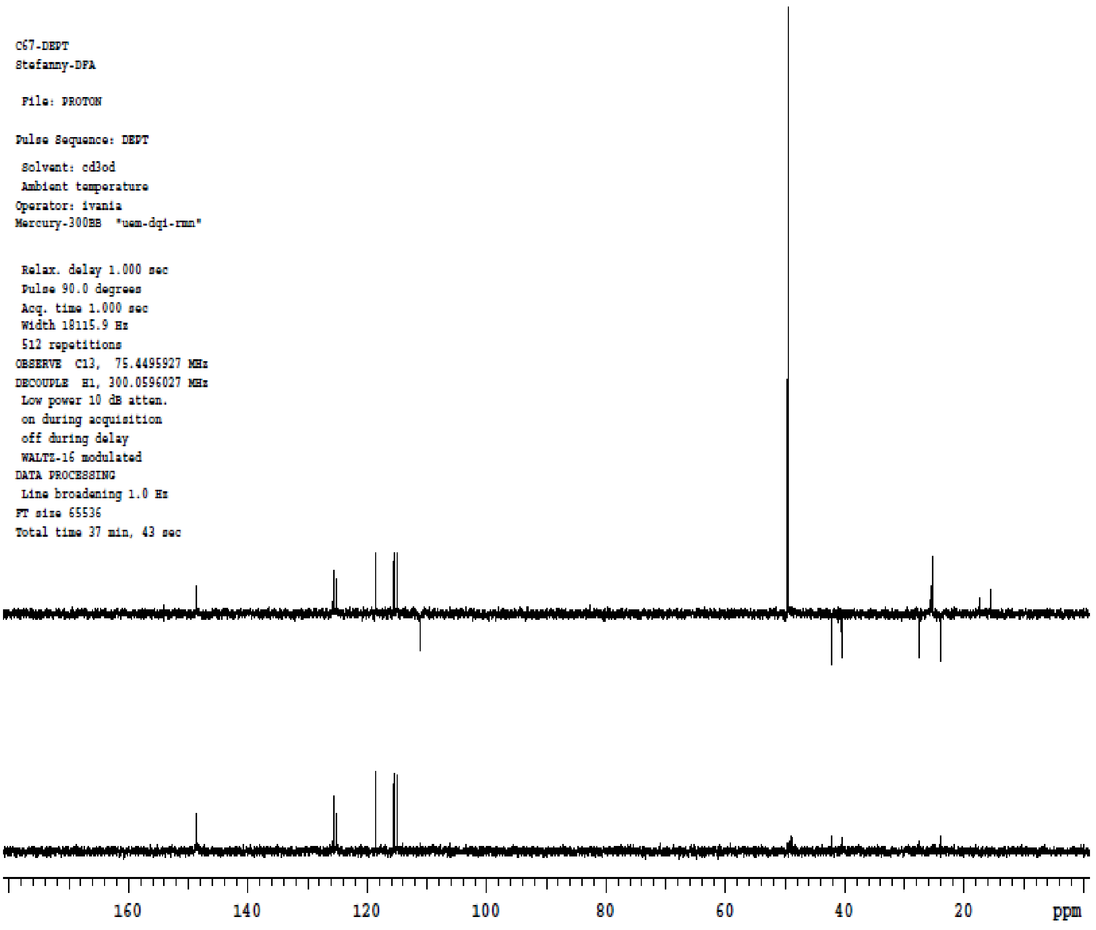

^{13}C DEPT NMR spectrum of the compound 4-Nerolidylcatechol (75.45 MHz, $CDCl_3$)

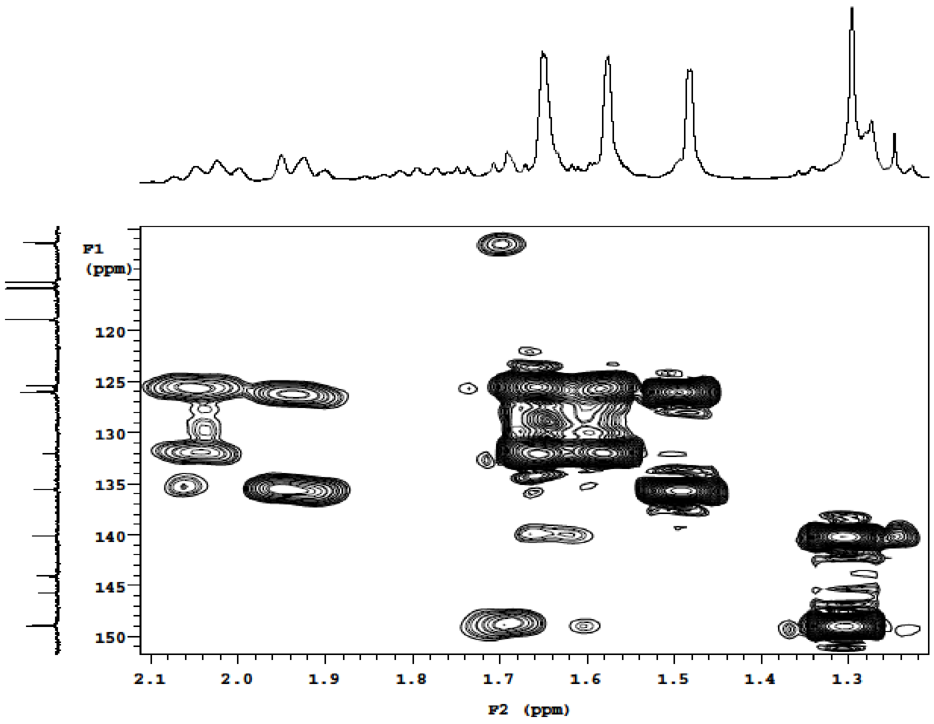

^{1}H–^{13}C HMBC spectrum of compound 4-NC (300 MHz, $CDCl_3$)

HPLC analysis of the oily extract from the residual fraction obtained from the leaves of Piper peltatum L.

The oily extract was evaluated under six different conditions at 4 mg/mL. The choice of mobile phase was based on the accessibility of the reagents used and on methodologies previously applied in the quantification of other Piperaceae species (Carrara, 2012).

A fingerprint of the oily fraction was obtained under the established conditions (Figure 12), following the evaluation parameters.

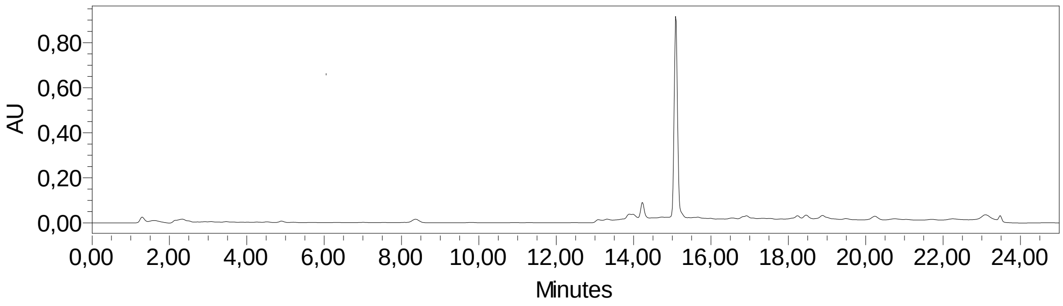

60% water–40% acetonitrile; 100% acetonitrile (10–20 min); 60% water–40% acetonitrile (20–25 min)

Identification of the fatty acids obtained from the hexane and hexane:dichloromethane fractions of the leaves of Piper peltatum L.

The fatty acids isolated from the hexane and hexane:dichloromethane fractions were identified by ^{1}H NMR analysis. Based on data from the literature, it was possible to confirm that they were fatty acids rather than other biologically relevant substances.

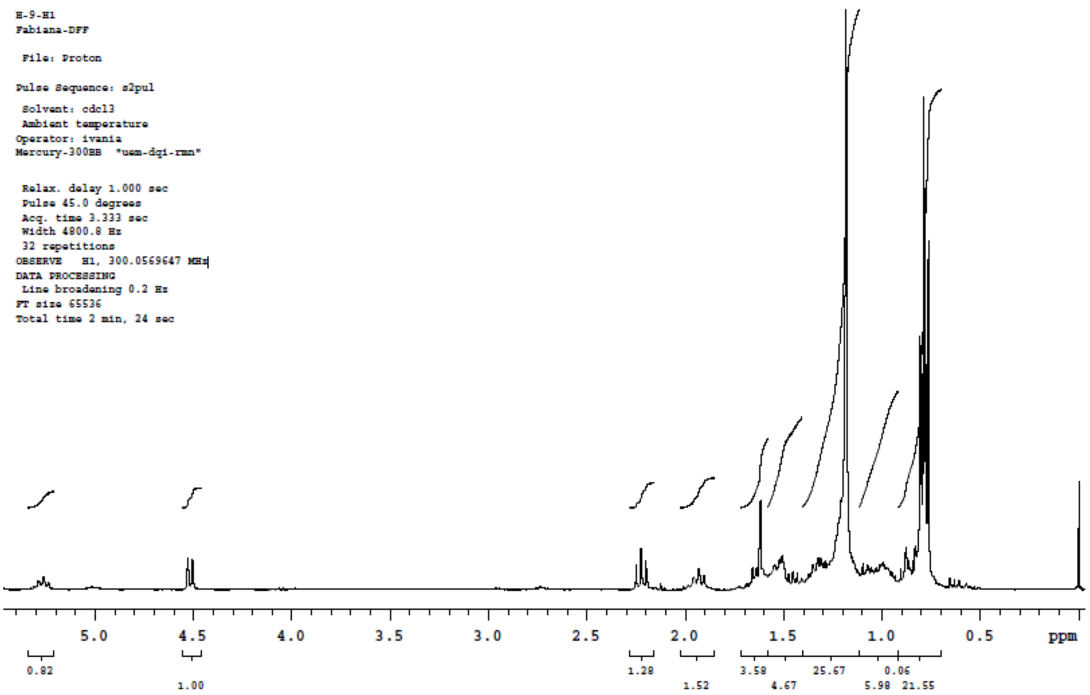

^{1}H NMR spectrum of the fatty acid isolated from the hexane fraction

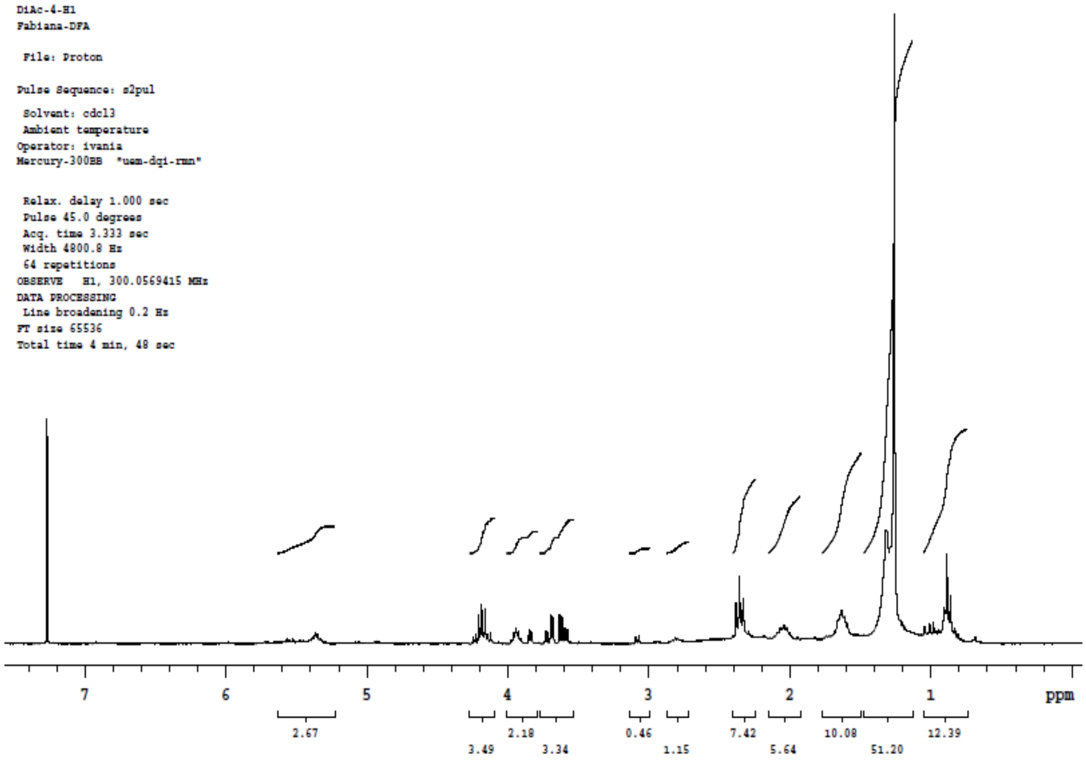

^{1}H NMR spectrum of the fatty acid isolated from the hexane:dichloromethane fraction
